# Evaluation of Lactose Oxidase as an Enzyme-Based Antimicrobial for Control of *L. monocytogenes* in Fresh Cheese

**DOI:** 10.3390/foods10071471

**Published:** 2021-06-25

**Authors:** Brenna Flynn, Dana deRiancho, Marie R. Lawton, Samuel D. Alcaine

**Affiliations:** Department of Food Science, Cornell University, Ithaca, NY 14853, USA; bf364@cornell.edu (B.F.); dld238@cornell.edu (D.d.); mrl255@cornell.edu (M.R.L.)

**Keywords:** *Listeria*, lactose oxidase, enzymes, queso fresco

## Abstract

*Listeria monocytogenes* is a ubiquitous pathogen that can cause morbidity and mortality in the elderly, immune compromised, and the fetuses of pregnant women. The intrinsic properties of fresh cheese—high water activity (aW), low salt content, and near-neutral pH—make it susceptible to *L. monocytogenes* contamination and growth at various points in the production process. The aim of this study was to investigate the ability of lactose oxidase (LO), a naturally derived enzyme, to inhibit the growth of *L. monocytogenes* in fresh cheese during various points of the production process. Lab-scale queso fresco was produced and inoculated with *L. monocytogenes* at final concentrations of 1 log CFU/mL and 1 CFU/100 mL. LO and LO sodium thiocyanate (TCN) combinations were incorporated into the milk or topically applied to the finished cheese product in varying concentration levels. A positive control and negative control were included for all experiments. When *L. monocytogenes* was inoculated into the milk used for the cheese-making process, by day 28, the positive control grew to above 7 log CFU/g, while the 0.6 g/L treatment (LO and LO + TCN) fell below the limit of detection (LOD) of 1.3 log CFU/g. In the lower inoculum, the positive control grew to above 7 log CFU/g, and the treatment groups fell below the LOD by day 21 and continued through day 28 of storage. For surface application, outgrowth occurred with the treatments in the higher inoculum, but some inhibition was observed. In the lower inoculum, the higher LO and LO-TCN concentrations (0.6 g/L) reduced *L. monocytogenes* counts to below the LOD, while the control grew out to above 7 log CFU/g, which is a >5 log difference between the control and the treatment. These results suggest that LO could be leveraged as an effective control for *L. monocytogenes* in a fresh cheese.

## 1. Introduction

*Listeria monocytogenes*, a ubiquitous intracellular pathogen [1], has the ability to proliferate at refrigeration temperature and can often contaminate products post-pasteurization, after which there is no further kill step to prevent ingestion of the pathogen by consumers. *L. monocytogenes* is of particular concern for certain dairy processors due to its ubiquitous presence in the environment and the physical properties of some ready-to-eat (RTE) dairy products such as fresh cheese [2].

The consumption of Hispanic-style cheeses is increasing in the United States [3]. This upsurge in popularity is likely due to the growth of the Hispanic population in the U.S., which reached 18.4% of the total population in 2019 [4]. Queso fresco, the most popular Hispanic-style fresh cheese [5], is a rennet coagulated cheese with intrinsic properties such as high moisture content, low salt content, and near-neutral pH [3], which make it susceptible to *L. monocytogenes* outgrowth when contaminated. According to the Centers for Disease Control and Prevention (CDC), there were eight foodborne outbreaks associated with *L. monocytogenes* contamination in cheese products between 2014 and 2018. The outbreaks included products such as quesarito (fresh cheese) curd [6], soft raw milk cheese [7], and most recently, queso fresco [8]. According to the National Outbreak Reporting System (NORS), there have been 5 outbreaks, 46 illnesses, 34 hospitalizations, and 2 deaths associated with *L. monocytogenes* contamination in queso fresco in the United States between 1971 and 2018 [9], which demonstrates that an effective control strategy for this pathogen is necessary.

There have been studies dedicated to controlling *L. monocytogenes* in queso fresco using a range of processing and antimicrobial treatments. High-hydrostatic-pressure processing (HPP) was found to immediately reduce *L. monocytogenes* contamination when queso fresco was treated at 600 MPa and held at a temperature of 20 °C for 3 min. However, this method is currently not cost-effective, significant wheying-off was observed, and the queso fresco was found to have different textural and rheological properties when compared to a control at 20 °C [10]. The efficacy of antimicrobial combinations of nisin, lauric arginate, and ɛ-polylysine [11], protective cultures [12], and phage endolysin PlyP100 [13] have all been explored to inhibit *L. monocytogenes* in queso fresco, demonstrating that finding an effective control method for this pathogen is a priority for the dairy industry. Consumers are currently moving toward a trend of desiring products with “clean labels” [14]. Therefore, finding effective antimicrobial agents that fall into the clean label category and are effective at preventing or reducing *L. monocytogenes* contamination in queso fresco would be beneficial to both consumers and producers by preventing further foodborne outbreaks and their associated human and financial costs.

Lactose oxidase (LO) is a naturally derived enzyme produced by a strain of mold called *Microdochium nivale* [15] that consumers would likely categorize under the clean-label category. Some cheeses are coagulated enzymatically with rennet [16], and therefore, consumers may be primed to view enzymes as a normal occurrence in dairy products. LO oxidizes lactose into lactobionic acid (LBA) [17] with the concurrent reduction of oxygen into hydrogen peroxide (H_2_O_2_) [15]. The structure of LBA consists of a polyhydroxy gluconic acid that is bonded to a glucose sugar (C_12_H_22_O_12_) [18]. It has been shown to inhibit counts of *L. monocytogenes* in milk when combined with other antimicrobial agents such as nisin and thymol [19]. Its effect on *L. monocytogenes* in a complex food matrix, such as cheese, when combined with hydrogen peroxide may yield promising results. H_2_O_2_ has been used in the dairy industry to preserve raw milk and breaks down into nontoxic compounds in solution [20]. It is approved for use at a concentration of 0.05% in solution for the milk used to produce several varieties of cheese, such as Colby, Cheddar, Swiss, and Emmentaler (21 Code of Federal Regulations (CFR) 184.1366). Treatment of fresh cheese with H_2_O_2_ was found to reduce *L. monocytogenes* counts by 3 log CFU/g when the cheese was exposed to a 10% solution for 5 s [21], and concentrations of 400 and 800 mg/L were bactericidal on *L. monocytogenes* within 24 h in a previous study [22].

LBA and H_2_O_2_ have been shown to be effective inhibitors of *L. monocytogenes* in dairy products, demonstrating that the production of these antimicrobial agents by LO itself may be an effective strategy to control *L. monocytogenes* in cheese. In a previous inhibition assay study, LO combined with sodium thiocyanate (TCN) was shown to inhibit the growth of *L. monocytogenes* [23]. TCN combined with hydrogen peroxide has been shown to be an effective activator of the lactoperoxidase system, which is a natural antimicrobial system present in raw milk [24,25]. In our previous study, LO and LO-TCN combinations were shown to inhibit *L. monocytogenes* in UHT skim milk and raw milk [26] (in review).

The efficacy of LO at inhibiting *L. monocytogenes* in a complex food matrix such as fresh cheese has not yet been investigated. The purpose of this study was to test multiple methods in which LO could be utilized, and the use of a laboratory-scale cheese model allowed various *L. monocytogenes* contamination scenarios and applications of LO and LO-TCN combinations to be explored. The first part of this study looks at the scenario in which the milk was contaminated with *L. monocytogenes* and looked at the efficacy of adding either LO or LO-TCN to the milk prior to making the cheese on the subsequent outgrowth of *L. monocytogenes* in the final cheese. The second part of this study investigates scenarios in which the surface of the queso fresco is contaminated by *L. monocytogenes* from the environment as well as the subsequent effect of LO and LO-TCN combinations application to the surface to prevent outgrowth. *L. monocytogenes* was inoculated onto the surface of the cheese at concentrations of either 200 CFU/g or 2 CFU/g. Then, LO and LO-TCN combinations were topically applied to the cheese surface. Optimal LO and LO-TCN concentrations for inhibiting *L. monocytogenes* growth in milk products were determined in our previous study [26] and tested during the cheese-making process. The addition of LO into the milk used for cheese making or topical application both represent potential control strategies that could be implemented by cheese makers to reduce the risk of *L. monocytogenes* outbreaks attributed to queso fresco and similar style cheeses.

## 2. Materials and Methods

### 2.1. Preparation of Listeria monocytogenes Inoculum

A cocktail of *L. monocytogenes* was prepared using five isolates of *L. monocytogenes* (Table 1). The strains, four from fresh cheese outbreaks and one a laboratory strain, were obtained from Dr. Martin Wiedmann’s Food Safety Laboratory at Cornell University (Ithaca, NY, USA). Each strain was streaked onto Brain Heart Infusion (BHI) agar (Beckton, Dickinson and Co., Franklin Lakes, NJ, USA) and incubated at 37 °C for 24 h. An individual colony of each strain from each streak plate was used to separately inoculate 5 mL of BHI broth. Broth cultures were incubated at 37 °C to obtain a concentration of OD = 1.00 (9 log CFU/mL). One mL of each culture was combined to produce a cocktail for the inoculation of milk or cheese samples. The *L. monocytogenes* cocktail was serially diluted in phosphate buffer saline (PBS) solution to the appropriate concentration for each experiment. The appropriate serial dilutions of the cocktail were enumerated on BHI agar for each experiment to confirm target inoculum concentrations.

### 2.2. Cheese Making

Laboratory-scale queso fresco was produced following previous methods with modifications dependent on the goal of each experiment that will be discussed as follows with modifications from a previously established method [12]. Pasteurized, homogenized milk was collected from the Cornell Dairy. Milk (600 mL) was aseptically added to 1 L sterile bottles (VWR International, Solon, OH, USA) and brought to 35 °C over the course of one hour in a water bath. The following were added to each bottle: 1875 μL of a 32% calcium chloride (CaCl_2_) solution (Dairy Connection Inc., Madison, WI, USA) and 78 μL of double-strength rennet (Chy-Max Extra; Chr. Hansen, Milwaukee, WI, USA). Each bottle was swirled to mix and poured into individual plastic cheese vats. The vats were incubated in a water bath at 35 °C for 65 min to promote curd formation. After 65 min, the curd was cut and allowed to heal for 10 min at 35 °C. Then, the temperature of the water bath was slowly brought to 43 °C over the course of 30 min, and the vats were incubated for 30 min at this temperature. Then, 60 mL of whey were removed from each cheese vat and replaced with 60 mL of a 0.16 g/mL NaCl solution. Each vat was returned to the water bath and incubated at a temperature set at 43 °C for 20 min. Then, the whey was drained using a sterile cheesecloth for 1 h. After drainage, 6 (±0.2) grams of cheese curd were aseptically transferred into 12-well plates (Corning, Inc., Corning, NY, USA). Then, the curd was pressed for 16 h overnight using cheese weights provided from the Cornell Dairy to produce a final curd weight of approximately 5 (±0.2) grams.

### 2.3. Application of LO and TCN for Inhibition of L. monocytogenes in the Milk 

Cheese was made as described in the earlier section with the following modifications. During the first step, the prepared *L. monocytogenes* cocktail was inoculated into the milk at final concentrations of approximately either 1 log CFU/mL or 1 CFU/100 mL. The bottles were inverted to ensure distribution of the inoculum throughout the milk. Following the one-hour incubation step to heat the milk, either LO (LactoYield, Chr. Hansen, Milwaukee, WI, USA) on its own or a combination of LO-TCN (VWR International, Solon, OH, USA) were added to each inoculated treatment. Separate experiments were performed using the LO treatment on its own or the LO-TCN combined treatment. Lactose oxidase was added into the milk to reach a final concentration of either 0.12 or 0.6 g/L in the milk. Sodium thiocyanate was added into the milk to reach a final concentration of 14 mg/L. A positive control with no added LO was used for each inoculum, and a negative control with no added LO or inoculum was used. The samples were stored at 6 °C for the entirety of the trial. For each of these LO and LO-TCN experiments, batches of cheese were made in triplicate to measure the pH using an edge meter (Hanna Instruments, Woonsocket, RI, USA) and water activity (aW) with an Aqualab meter (METER Group, Pullman, WA, USA) in duplicate throughout the storage conditions of the treatment following the same sampling period as microbiological analysis.

### 2.4. Enumeration of Samples for Microbiological Analysis

For each trial, cheese samples were enumerated for microbiological analysis using the same method. Samples were enumerated on days 0, 1, 2, 4, 7, 14, 21, and 28 for *L. monocytogenes* counts. Each sample was aseptically added to a Whirl-Pak filter bag (Nasco, Fort Atkinson, WI, USA), and a 1:10 (*w/v*) dilution using PBS was performed. The samples were digested at normal speed for 60 s using a Seward Stomacher 400 Lab Blender Series (VWR International, Solon, OH, USA). The digested samples were diluted in 9 mL PBS blanks to the appropriate dilution and vortexed. Then, dilutions were spread-plated on Modified Oxford Agar (MOX) plates in duplicate and incubated for 48 h at 37 °C. *L. monocytogenes* counts were taken after 48 h of incubation. Each experiment was performed at least in triplicate.

### 2.5. Preparation of LO and TCN Solutions for Surface Application

The LO and TCN solutions for surface application were prepared as follows. A 16% lactose solution was combined with 5 mL of a 0.24 g/L LO solution and filter sterilized through a 0.20 μm surfactant-free cellulose acetate filter (Corning Inc., Corning, NY, USA). A 1.2 g/L LO solution was combined with a 16% lactose solution and filter sterilized through a 0.20 μm surfactant-free cellulose acetate filter (Corning Inc.). Final concentrations of 0.12 g/L LO 8% lactose and 0.6 g/L LO 8% lactose in solution were produced when combined. A 14 mg/L solution of TCN was made and filter sterilized through a 0.20 μm surfactant-free cellulose acetate filter (Corning Inc., Corning, NY, USA). Each treatment was stored at 6 °C for the entirety of the trial. For each of these LO and LO-TCN surface experiments, batches of cheese were made to measure the pH and aW in duplicate throughout the storage conditions of the treatment following the same sample period as microbiological analysis.

### 2.6. Surface Application of LO and TCN for Inhibition of L. monocytogenes on Cheese Surface

Laboratory-scale cheese was produced using the method described in the cheese-making section. Following the overnight press, 100 µL of *L. monocytogenes* cocktail was inoculated on the surface of the cheese to obtain final concentrations of approximately 200 and 2 CFU/g. Cheese with no inoculum was included as a negative control. The inoculum was allowed to attach for approximately 45 min.

For each experiment, the following treatments were added to the cheese. For the LO treatments, 100 μL of either the 0.12 g/L LO or 0.6 g/L LO and 8% lactose solution was dispensed onto the surface of the cheese. For the TCN treatment, the respective LO solution was dispensed onto the surface of the cheese with 100 μL of TCN solution. For the negative and positive control, 100 µL of sterile MilliQ water was dispensed onto the cheese. Cheese samples were enumerated as described in the previous section.

### 2.7. Statistical Analysis

Each experiment was repeated in triplicate. All statistical analyses were performed using R software (Version 3.5.2, R Development Core Team, Vienna, Austria). Analysis of Variance and Tukey’s Honest Significant Difference tests were performed at each time point to determine log differences in *L. monocytogenes* counts between all treatments and the positive control. The same tests were performed at each time point to determine differences in pH and aW values between all treatments and the negative control.

## 3. Results and Discussion

### 3.1. Impact of LO Application in the Milk during the Cheese-Making Process on L. monocytogenes Outgrowth

Final concentrations of 0.12 and 0.6 g/L LO in solution were added to the milk during the cheese-making process to determine their efficacy against *L. monocytogenes* in laboratory-scale queso fresco. *L. monocytogenes* inoculum was added into the milk to achieve concentrations of 1 log CFU/mL and 1 CFU/100 mL, representing variable contamination scenarios. *L. monocytogenes* can contaminate bulk tank milk samples from non-aseptic sampling of the cow udder or milking equipment [27], where it may then proliferate during the cheese-making process. This experiment aimed to determine the antimicrobial effect of LO on *L. monocytogenes* contamination that could occur with the incoming milk product used for making cheese.

**Figure 1:**
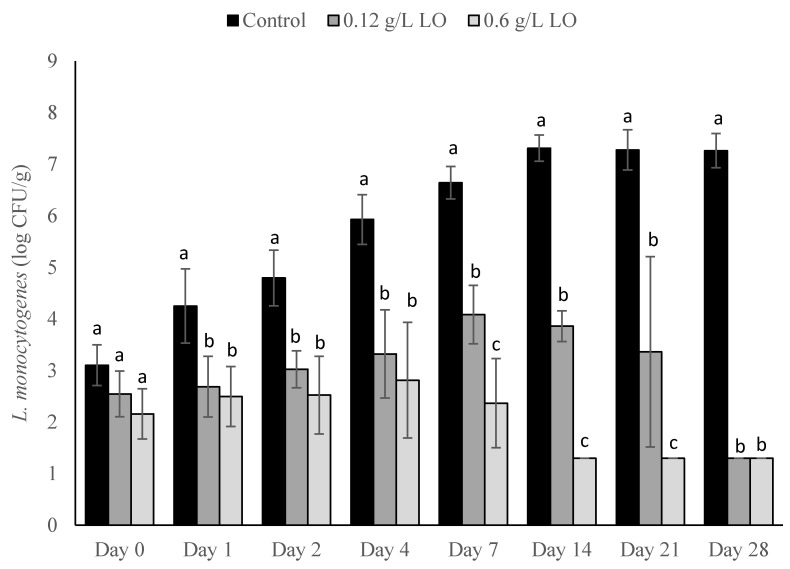
*L. monocytogenes* counts presented as log_10_ CFU/g in pasteurized queso fresco that was inoculated at 1 log_10_ CFU/mL in the milk used for making the cheese and treated with lactose oxidase (LO) during storage at 6 °C. Numbers on the treatment label indicate the concentration of LO solution (g/L). Bars with different letters indicate significant differences (*p* < 0.05) between treatments on the same day. For counts lower than the limit of detection, a value of 1.3 log_10_ CFU/g was used. Error bars represent the SD.

At *L. monocytogenes* inoculation levels of 1 log CFU/mL into the milk, both the 0.12 g/L LO and 0.6 g/L LO treatments showed less outgrowth of *L. monocytogenes* than the control (Figure 1). Inoculation of the milk at a concentration of 1 log CFU/mL resulted in a final concentration of approximately 3 CFU/g in the final cheese control on day 0 of storage. On day 0, there was no significant difference in *L. monocytogenes* counts between the treatments and the control. However, significant differences (*p* < 0.05) between the treatment groups and the control were observed starting at day 1 of storage and continued throughout the entire trial. Both treatments grew out slightly from the initial inoculum, with the 0.12 g/L LO treatment reaching a maximum of 4 log CFU/g on day 7 of storage and the 0.6 g/L LO treatment reaching a maximum of 2.8 log CFU/g on day 4 of storage. After day 7 of storage, the *L. monocytogenes* counts dropped in the treatment groups, while the control presented outgrowth. On days 14, 21, and 28, the control grew to above 7 log CFU/g, while the 0.6 g/L LO treatment fell below the limit of detection (LOD) of 1.3 log CFU/g. The *L. monocytogenes* counts for the 0.12 g/L treatment dropped starting on day 14 and fell below the LOD on day 28 of storage. On days 14, 21, and 28, a listericidal effect was observed using the 0.6 g/L LO treatment, and on day 28, the same effect was present with the 0.12 g/L treatment because the treatment groups were reduced to levels below the initial inoculum.

**Figure 2:**
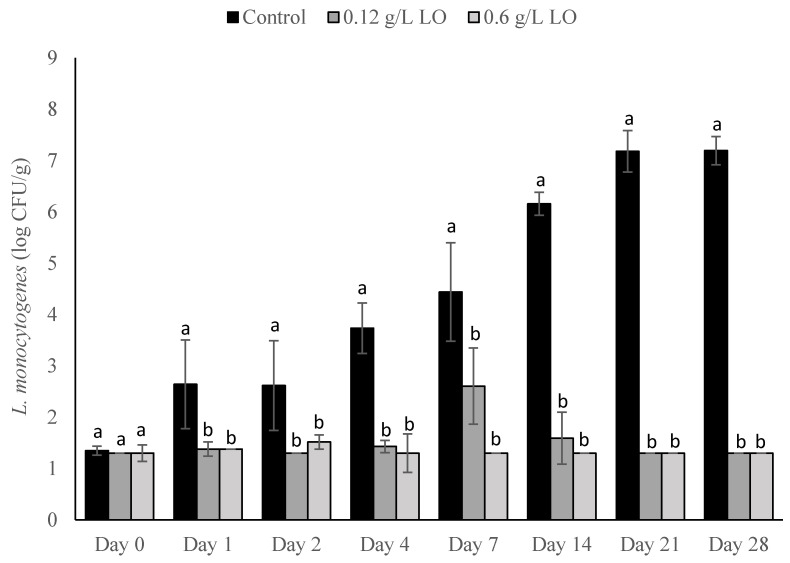
*L. monocytogenes* counts presented as log_10_ CFU/g in pasteurized queso fresco that was inoculated at 1 CFU/100 mL in the milk used for making the cheese and treated with lactose oxidase (LO) during storage at 6 °C. Numbers on the treatment label indicate the concentration of LO solution (g/L). Bars with different letters indicate significant differences (*p* < 0.05) between treatments on the same day. For counts lower than the limit of detection, a value of 1.3 log_10_ CFU/g was used. Error bars represent the SD.

When *L. monocytogenes* was inoculated into the milk at a concentration of 1 CFU/100 mL, counts for both treatments and the control did not reach above the LOD (1.3 log CFU/g) until day 1 of storage (Figure 2). Both treatments inhibited *L. monocytogenes* outgrowth throughout the entirety of the trial. The 0.12 g/L treatment reached a maximum concentration of approximately 2.5 CFU/g on day 7 of storage, while the control reached a level above 4 log CFU/g on that day. Except for day 7, both treatment groups averaged below 2 CFU/g throughout the trial. On days 21 and 28, the control reached levels above 7 log CFU/g, while both treatment groups fell below the LOD.

These results suggest that the production of H_2_O_2_ by LO is sufficient to inhibit *L. monocytogenes* counts at different concentrations in queso fresco. A previous study [23] tested the effect of LO as an antimicrobial against *L. monocytogenes* using an overlay inhibition assay, and microbial inhibition was observed. Catalase was added to the treatments, and then, *L. monocytogenes* growth occurred, suggesting that a primary cause of bacteria inhibition was the production of H_2_O_2_ by the LO reaction in solution. In both of our contamination scenarios, LO inhibited or reduced *L. monocytogenes* outgrowth throughout the entire trial. The application of hydrogen peroxide to reduce *L. monocytogenes* counts has been shown to be effective in a variety of products such as mung bean sprouts [28], organic fresh lettuce [29], milk [22], and high-moisture soft cheese [21]. Robinson and D’Amico [21] found that the treatment of queso fresco with a 10% H_2_O_2_ solution significantly reduced *L. monocytogenes* counts by 2.27 log CFU/g in the first 30 min of treatment and by approximately 0.5 log CFU/g after the first 24 h of storage, with no regrowth after storage. Kozak [22] found that in milk, a 400 mg/L H_2_O_2_ solution was bactericidal against *L. monocytogenes*. Our results utilizing H_2_O_2_, a product of LO in dairy applications, as an antimicrobial for *L. monocytogenes* growth are consistent with these previous studies.

### 3.2. Impact of LO Additon to Milk Used to Produce Queso Fresco on pH

Significant differences were observed starting at day 1 of storage between the LO treatment groups and the control, and these differences continue until day 28 of the experiment (Table 2). By day 2 of the experiment, the control, 0.12 g/L LO, and 0.6 g/L LO treatment groups were all statistically significant from one another. This trend lasted throughout the entirety of the trial after day 2. By the end of the trial, the pH of the treatment groups were 0.57 and 1.02 pH units lower than the control for the 0.12 and 0.6 g/L treatments, respectively.

The drop in pH observed in this study is likely due to the oxidation of lactose into LBA from the addition of LO into the queso fresco product. In this cheese-making process, no starter cultures were added, and while there may be some background lactic acid bacteria in the pasteurized milk, the steady pH of the control cheese (Table 2) suggests that bacterial acidification is not the driver of the pH drop in the samples. LBA is comprised of one galactose molecule that is attached to one molecule of gluconic acid via an ether-like linkage. The use of LBA has been investigated in the dairy industry as a key ingredient in novel dairy technologies [30]. A study showed that LBA exhibited antimicrobial properties against both Gram-negative and Gram-positive bacteria in pasteurized whole milk [31]. Our previous study [26] showed that a pH drop was exhibited in milk at low concentrations from the addition of LO. Our current results are consistent with these data. Further sensory analysis should be explored to determine consumer perception of the pH drop in queso fresco. Sensory analysis was performed with the addition of LBA to whole milk [31], and it delayed the deterioration of sensory qualities in the milk. Therefore, although a pH drop is present in our study, there could be a potential to use LO as a biopreservation method in further studies to reduce spoilage in cheese as well as its application to reduce pathogenic growth. Furthermore, the pH reduction over time may also explain why *L. monocytogenes* counts fell below the LOD in the microbiological study by day 14 of storage for the higher concentration treatment (0.6 g/L LO) and by day 28 of storage for the lower concentration treatment (0.12 g/L LO).

A previous study [32] evaluated the effect of different acids on the outgrowth of *L. monocytogenes* in queso fresco. Depending on the acid type, moisture content, and salt content of the cheese, the addition of certain acids inhibited *L. monocytogenes* outgrowth over an 8-week period. When acetic acid and propionic acid were added to the cheese to produce final pH values of 5.25–5.75, at all moisture content and salt content percentages, there was no weekly growth of *L. monocytogenes* in the cheese. When lactic acid was added, outgrowth was observed at pH levels above 5.25, with moisture content of 50–56% and an NaCl concentration of 1.25%. In our study, the pH of the 0.6 g/L LO treatment dropped to 5.69–5.38 (day 14 and day 28), and *L. monocytogenes* counts fell below the LOD in both challenge studies. When the pH reached 5.83 on day 28 of storage, the 0.12 g/L LO treatment also fell below the LOD. Thus, it is possible that the drop in pH due to LBA production, particularly in the 0.6 g/L treatment, also played a role in controlling *L. monocytogenes* outgrowth.

### 3.3. Impact of LO Addition into the Milk during the Cheese-Making Process on Water Activity 

The water activity of the control and treatment groups remained consistent throughout the entirety of the experiment (Table 3). The aW remained at 0.97 for all treatments throughout the whole trial; therefore, there was no significant difference between the control and the treatment groups. The standard aW value for queso fresco is 0.98 [33]; our results are consistent with this standard. Therefore, from the results provided by this study, the addition of LO to milk does not influence the water activity of queso fresco.

### 3.4. Impact of LO-TCN into the Milk during the Cheese-Making Process on L. monocytogenes

LO addition into the milk alone showed antimicrobial properties on *L. monocytogenes* in queso fresco over time. Since LO alone was shown to have a listericidal effect, we then explored the addition of TCN combined with LO to investigate if this combination had further antimicrobial properties. In our previous study [26], LO-TCN combinations inhibited *L. monocytogenes* in raw milk more effectively than LO alone. Therefore, these concentrations were utilized to investigate the effect against *L. monocytogenes* in lab-scale queso fresco.

**Figure 3:**
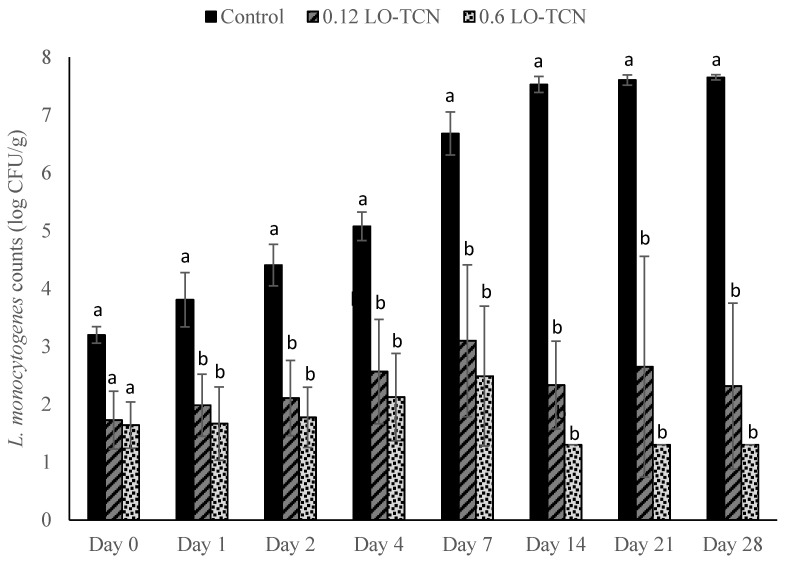
*L. monocytogenes* counts presented as log_10_ CFU/g in pasteurized queso fresco that was inoculated at 1 log_10_ CFU/mL in the milk used for making the cheese and treated with lactose oxidase (LO) and 14 mg/L sodium thiocyanate (TCN) during storage at 6 °C. Numbers on the treatment label indicate the concentration of LO solution (g/L). Bars with different letters indicate significant differences (*p* < 0.05) between treatments on the same day. For counts lower than the limit of detection, a value of 1.3 log_10_ CFU/g was used. Error bars represent the SD.

**Figure 4:**
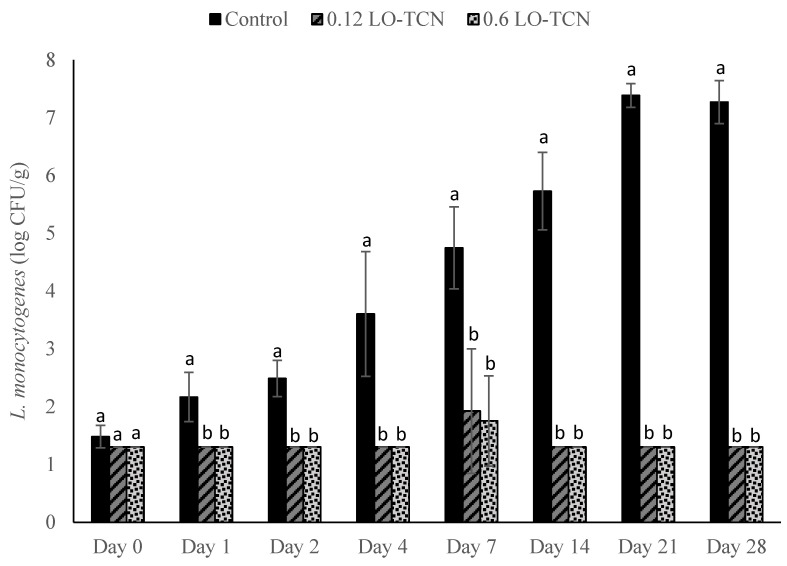
*L. monocytogenes* counts presented as log_10_ CFU/g in pasteurized queso fresco that was inoculated at 1 CFU/100 mL in the milk used for making the cheese and treated with lactose oxidase (LO) and 14 mg/L sodium thiocyanate (TCN) during storage at 6 °C. Numbers on the treatment label indicate the concentration of LO solution (g/L). Bars with different letters indicate significant differences (*p* < 0.05) between treatments on the same day. For counts lower than the limit of detection, a value of 1.3 log_10_ CFU/g was used. Error bars represent the SD.

LO-TCN treatments displayed a similar level of growth as in our LO experiments when the milk during the cheese-making process was inoculated with an *L. monocytogenes* cocktail at a final concentration of 1 log CFU/mL (Figure 3). Except for day 0, during the entirety of the trial, both the low (0.12) and high (0.6) g/L LO treatments displayed significant differences *(p* < 0.05) in *L. monocytogenes* outgrowth in comparison to the control. The 0.12 and 0.6 g/L LO treatments reached the highest level of outgrowth on day 7, where they reached levels of approximately 3.1 and 2.5 log CFU/g, respectively. These levels were reduced throughout the rest of the trial. By day 14 of storage and continuing to day 28, the *L. monocytogenes* counts for the 0.6 g/L treatment dropped below the LOD, while the control displayed outgrowth to above 7 log CFU/g. At the lower challenge level of 1 CFU/100 mL, supplementation with TCN resulted in lower *L. monocytogenes* outgrowth (Figure 4) in comparison to treatment with LO alone. The treatment groups (0.12 and 0.6 g/L LO-TCN) fell below the LOD on all days except for day 7. By day 21 and day 28 of the experiment, the control grew out to levels above 7 log CFU/g, while the treatment groups only reached the LOD of 1.3 log CFU/g.

The addition of TCN into raw milk activates the lactoperoxidase system (LPS), which is a natural antimicrobial system that is present in raw milk. The LPS is comprised of three components: hydrogen peroxide, thiocyanate, and lactoperoxidase. Lactoperoxidase catalyzes the oxidation of thiocyanate by hydrogen peroxide, which generates compounds such as hypothiocyanite ions, which act as antimicrobials. The efficacy of the LPS varies and relies on the concentration of thiocyanate and hydrogen peroxide. Thiocyanate in raw milk is present in close to optimal concentrations, but hydrogen peroxide must be added by other means, such as the addition of LO, to optimize the effect of the LPS in dairy products [24].

The concentration of lactoperoxidase in bovine raw milk is 1.2–16.2 ppm. In pasteurized milk, lactoperoxidase retains approximately 70% of its residual activity when pasteurized at 72 °C for 15 s, and complete deactivation of the enzyme occurs when milk is pasteurized at 80 °C for 15 s [34]. The results for TCN supplementation at both the high (1 log CFU/mL) and low (1 CFU/100 mL) inoculum were similar to that of the LO trials. Therefore, either the LPS produced a slight antimicrobial effect against *L. monocytogenes* in these trials, especially in the lower inoculum, or the inhibition was due to H_2_O_2_ production by LO.

### 3.5. Impact of LO-TCN Addition to Milk during the Cheese-Making Process on pH

Significant differences in pH began on day 2 of the trial, and the pH was reduced throughout the entirety of the experiments (Table 4). By day 28, the pH of the 0.12 g/L treatment dropped to 5.78, while the 0.6 g/L treatment group dropped to 5.45, and the pH of the control remained at 6.33. The production of LBA, as discussed previously, is likely the reason for this pH reduction. The addition of TCN did not prevent a pH reduction throughout the trial.

### 3.6. Impact of LO-TCN Addition to the Milk during the Cheese-Making Process on Water Activity

The data in Table 5 demonstrate that no significant difference *(p >* 0.05) was displayed throughout the entirety of the trial using 0.12 and 0.6 g/L LO-TCN combinations. The aW of the control and treatments fell between 0.97 and 0.98 units during the entire trial, which is close to the 0.98 industry standard. Therefore, the addition of LO-TCN combinations does not influence water activity, which is promising for further sensory analysis.

### 3.7. Impact of Application of LO and LO-TCN Combinations on the Surface of the Cheese on L. monocytogenes Growth 

**Figure 5:**
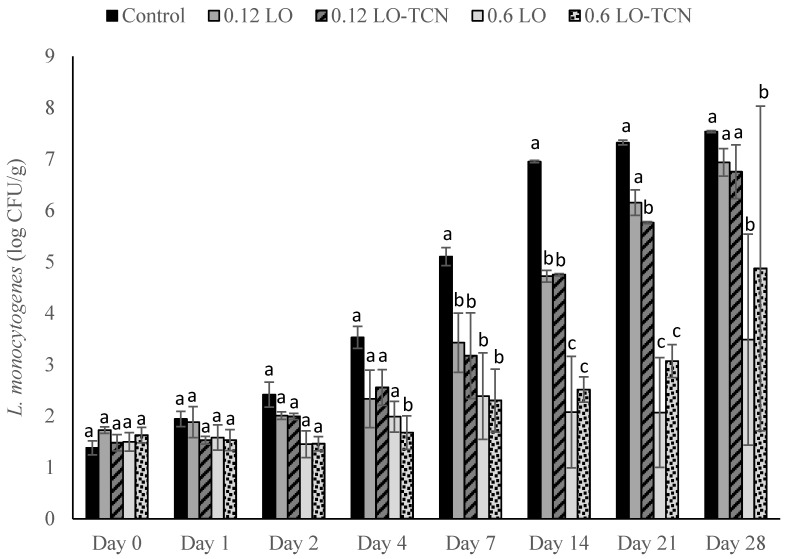
*L. monocytogenes* counts presented as log_10_ CFU/g in pasteurized queso fresco that was inoculated at 200 CFU/g on the surface of the cheese and topically treated with lactose oxidase (LO) and 14 mg/L sodium thiocyanate (TCN) during storage at 6 °C. Numbers on the treatment label indicate the concentration of LO solution (g/L). Bars with different letters indicate significant differences (*p* < 0.05) between treatments on the same day. For counts lower than the limit of detection, a value of 1.3 log_10_ CFU/g was used. Error bars represent the SD.

*L. monocytogenes* may contaminate dairy products by contaminating the raw materials (i.e., contaminated or improperly pasteurized milk) used to make products or through post-pasteurization contamination from the processing environment [2]. The second part of this study aimed to explore surface contamination with *L. monocytogenes* of queso fresco from the processing environment. Cheese was made in the laboratory-scale fashion; *L. monocytogenes* was inoculated onto the surface of the cheese at concentrations of 200 CFU/g or 2 CFU/g, and solutions of lactose oxidase or lactose oxidase with sodium thiocyanate were topically applied to examine their effect on surface *L. monocytogenes* contamination. When samples were inoculated at an *L. monocytogenes* concentration of 200 CFU/g, significant differences between the treatment groups and the control were not observed until d 4 of storage (Figure 5). These differences remained significant throughout the rest of the trial. The greatest antimicrobial inhibition was observed on day 14 of storage, when the 0.12 g/L treatments fell to below 5 log CFU/g and the 0.6 g/L treatment groups fell below 3 log CFU/g, while the control grew out to above 6.5 log CFU/g. Average levels of outgrowth did not reach that of the control in the 0.6 g/L LO and 0.6 g/L LO-TCN treatments throughout the trial; however, *L. monocytogenes* counts increased in the treatment groups throughout storage.

High standard deviations occurred for both 0.6 g/L treatments on day 28 of storage. This was due to the variation between trials for both treatment groups. The 0.6 g/L treatment had levels of growth that were below the LOD for one trial, and they reached 3.8 and 5.4 log CFU/g for the other two trials. The 0.6 g/L LO-TCN treatment had levels of growth that reached 6 to 7 log CFU/g for two of the trials but fell below the LOD for one trial. These large discrepancies could be due to variability in the way LO and TCN treatments were topically applied to each 5 g cheese sample enumerated per day of storage. When applying LO treatments in the food industry, a more methodical approach to surface application, such as spraying, could be used to ensure the entire surface of the cheese is covered with the treatment. Furthermore, there was no significant difference between the topical application of LO alone and LO in combination with TCN in these trials.

**Figure 6:**
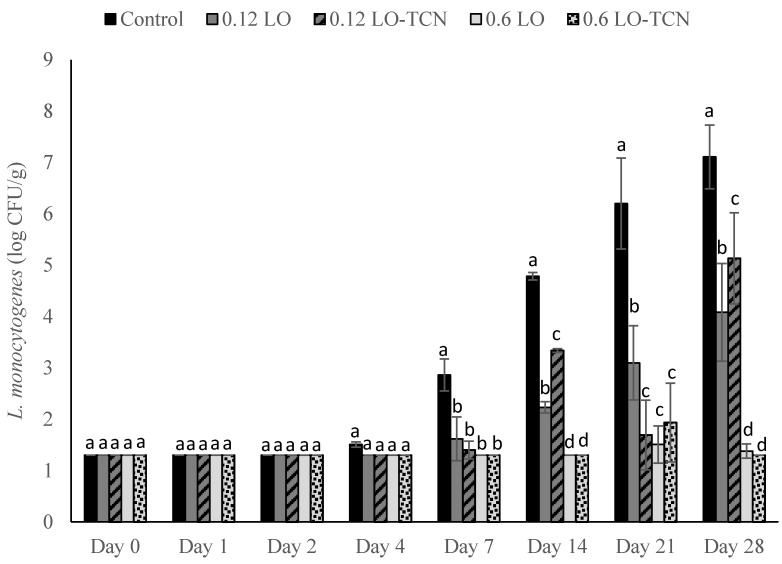
*L. monocytogenes* counts presented as log_10_ CFU/g in pasteurized queso fresco that was inoculated at 2 CFU/g on the surface of the cheese and topically treated with lactose oxidase (LO) and 14 mg/L sodium thiocyanate (TCN) during storage at 6 °C. Numbers on the treatment label indicate the concentration of LO solution (g/L). Bars with different letters indicate significant differences (*p* < 0.05) between treatments on the same day. For counts lower than the limit of detection, a value of 1.3 log_10_ CFU/g was used. Error bars represent the SD.

No *L. monocytogenes* growth occurred until day 4 of storage in the low inoculum challenge (Figure 6). Starting on day 4 of the trial, the control exhibited outgrowth, while the treatments inhibited the growth of *L. monocytogenes*. The 0.12 g/L LO and 0.12 g/L LO-TCN treatments grew out from the original inoculum; however, they still exhibited significant differences (*p* < 0.05) from the control throughout the storage period and fell approximately 3 log CFU/g below the control on day 21 of storage before growing out on day 28. The 0.6 g/L LO and 0.6 g/L LO-TCN treatments fell below the LOD on all days except for days 21 and 28 of storage, and on days 21 and 28, only slight outgrowth occurred. By day 28 of storage, these treatments fell more than 5 log CFU/g below the control.

The purpose of this experiment was to study if the outgrowth due to surface contamination by *L. monocytogenes* could be controlled with the topical application of LO and LO-TCN combinations in queso fresco. At higher inoculum levels, the outgrowth of *L. monocytogenes* occurred in both the control and the treatment groups, with some inhibition by LO treatment. While we were applying concentrations of 0.12 and 0.6 g/L LO and 14 mg/L TCN concentrations onto the surface of the cheese, the final concentration of components in the cheese is much lower, and thus, it is much lower than the LO and TCN concentrations we used in the earlier experiments where the components were directly added to the milk. The total amount of LO and TCN used in the cheese was calculated as follows. Since each cheese sample was pressed in its own well in a 12-well plate, the use of these wells as cheese molds produced uniform cheeses that had a surface area of 3.8 cm^2^. When solutions of 0.6 and 0.12 g/L LO were topically applied, the final concentration of LO solutions was 1.58 × 10^−5^ and 3.16 × 10^−6^ g LO per cm^2^ of cheese, respectively. The TCN was added at a concentration of 3.68 × 10^−4^ mg TCN/cm^2^ of cheese. In the final cheese product, LO concentrations were incorporated at 1.20 × 10^−5^ (0.6 g/L LO solution addition) and 2.40 × 10^−6^ (0.12 g/L LO solution addition) g LO per g of cheese. The TCN solution was incorporated at 2.80 × 10^−4^ mg TCN per g of cheese when a 14 mg/L solution was topically applied.

While surface application means there is more oxygen available for LO, because of its lower concentration in the cheese, this would have resulted in lower total hydrogen peroxide production by LO in comparison to the treatments where LO was added directly to the milk. Still, at the lower challenge inoculum (2 CFU/g), outgrowth was inhibited by the 0.6 g/L LO and LO-TCN combinations on the surface of the cheese.

Additional supplementation with lactoperoxidase enzyme in pasteurized milk may increase the antimicrobial effect of the LPS, as displayed by a previous study that used LO and the LPS to inhibit spoilage in milk [35]. Future studies should explore the optimization of LO, TCN, and LPS levels to produce the greatest antimicrobial inhibition of *L. monocytogenes* in a laboratory-scale queso fresco. Furthermore, future studies should also explore these same concentrations topically applied at concentrations of 0.12 and 0.6 g/L LO total in the cheese.

### 3.8. Impact of LO and LO-TCN Combinations on Cheese pH 

The 0.12 g/L LO and 0.12 g/L LO-TCN combination did not display significant differences *(p >* 0.05) in pH from the control (Table 6), suggesting that the production of LBA was minimal for these treatments when compared to the experiments that added LO into the milk (Table 4). The pH of the 0.6 g/L LO and 0.6 g/L LO-TCN combination displayed significant differences from the control by day 7 of storage, and this drop continued until day 28. By the end of the trial, both treatments were more than 0.20 pH units below the control. The reduced pH of the 0.6 g/L LO and 0.6 g/L LO-TCN treatments may have caused greater antimicrobial reduction when compared to the 0.12 g/L LO and 0.12 g/L LO-TCN combinations.

When compared to the treatments where LO and TCN were added into the milk, there was not as large of a pH reduction when the treatments were applied topically. When the LO treatments were added into the milk used for the cheese-making process, the pH was reduced to 5.38 and 5.83 for the 0.6 and 0.12 g/L treatments, respectively. When treatments were topically applied, the pH remained above 6.0 throughout the entire trial for all treatments. This relatively small decrease in pH may be attributed to the lower concentrated solution of LO applied to the cheese surface discussed previously, and consequently, less production of LBA over time. When various organic acids were added to queso fresco to produce a pH of 6.0 in a previous study, *L. monocytogenes* was able to grow at all moisture and NaCl levels [33].

Outgrowth was inhibited when the lower inoculum was applied to the surface of the cheese and the 0.6 g/L LO and LO-TCN combinations were topically applied, although the pH remained above 6.0 (Figure 6). Outgrowth was also slowed with the 0.12 g/L LO and LO-TCN treatments. This suggests that either of the antimicrobial products, H_2_O_2_ and LBA, of LO were sufficient to inhibit *L. monocytogenes* growth at low levels of incidental contamination on a cheese surface.

### 3.9. Impact of LO and LO-TCN Combinations on the aW of Queso Fresco

The aW of the treatments when compared to the control did not change throughout the entire trial (Table 7). The aW remained between 0.97 and 0.98 for both the control and the treatments. Therefore, the LO and LO-TCN combinations do not change the water activity of the cheese over time when topically applied.

## 4. Conclusions

In this study, we explored the effect of lactose oxidase on its own and in combination with TCN as a method to control *L. monocytogenes* outgrowth in a laboratory-scale fresh cheese model. We first explored the inoculation of *L. monocytogenes* and the addition of LO and LO-TCN combinations into the incoming raw milk. We determined that LO and LO-TCN combinations inhibit the growth of *L. monocytogenes* in a concentration-dependent fashion. We determined that LO is effective as a listericidal control method with both a high (1 log CFU/mL) and a low (1 CFU/100 mL) inoculum in the milk used during the cheese-making process. These treatments did cause a significant change in the pH of the cheese, which may affect sensory analysis and should be explored further in future studies.

Then, we explored the efficacy of LO and LO-TCN combinations as a topical application for the surface contamination of queso fresco and determined that LO showed efficacy in low-level contaminant scenarios. The level of initial surface contamination by *L. monocytogenes* in the real world is difficult to know definitely, and it obviously varies by the conditions of the event. Our results suggests that the surface application of LO, with or without TCN supplementation, could be useful for controlling incidental low-level *L. monocytogenes* from the environment onto the surface of the cheese.

Overall, the aim of this study was to explore a novel method to control *L. monocytogenes* outgrowth in a laboratory-scale queso fresco to improve the safety of high-risk cheeses. In conjunction with good hygienic practices, LO represent a novel tool that cheesemakers could use to improve the safety of their cheeses. Further research is needed to optimize the use of LO and understand the potential synergies with other antimicrobials that can be used to control *L. monocytogenes*.

## Figures and Tables

**Table 1:** Strains of *Listeria monocytogenes* used to produce a cocktail used for the inoculation of milk samples.

ID	Outbreak	Source Type	Source Site	Isolate Date	Serotype
FSL-X1-0001	Lab Strain 10403S	-	-	-	1/2a
FSL-R9-5621	2012 Ricotta Cheese	Food	Cheese	19 June 2012	1/2a
FSL-R9-5623	2013 Semi Fresh Style Cheese	Human	Placenta	29 May 2013	4b
FSL-R9-5625	2014 Soft Cheese	Human	Blood	6 July 2014	4b
FSL-R9-5624	2013 Queso Fresco	Human	Blood	14 August 2013	1/2b

**Table 2:** pH (±SD) of pasteurized queso fresco treated with lactose oxidase in the milk used for making the cheese during storage at 6 °C.

Time (Days)								
Treatment ^1^	0	1	2	4	7	14	21	28
Control	6.36 ± 0.08 ^a^	6.32 ± 0.03 ^a^	6.29 ± 0.04 ^a^	6.31 ± 0.01 ^a^	6.32 ± 0.02 ^a^	6.33 ± 0.02 ^a^	6.30 ± 0.00 ^a^	6.40 ± 0.10 ^a^
0.12 g/L LO	6.29 ± 0.05 ^a^	6.21 ± 0.04 ^b^	6.18 ± 0.01 ^b^	6.15 ± 0.04 ^b^	6.08 ± 0.04 ^b^	5.94 ± 0.02 ^b^	5.93 ± 0.10 ^b^	5.83 ± 0.07 ^b^
0.6 g/L LO	6.25 ± 0.03 ^a^	6.14 ± 0.04 ^b^	6.02 ± 0.03 ^c^	5.94 ± 0.07 ^c^	5.83 ± 0.06 ^c^	5.69 ± 0.05 ^c^	5.59 ± 0.07 ^c^	5.38 ± 0.15 ^c^

^a,b,c^ Means within a column with different letters are significantly different (*p* < 0.05) between treatments. ^1^ LO = lactose oxidase; *n* = 8.

**Table 3:** aW (±SD) of pasteurized queso fresco treated with lactose oxidase in the milk used for making the cheese during storage at 6 °C.

Time (Days)								
Treatment ^1^	0	1	2	4	7	14	21	28
Control	0.97 ± 0.00 ^a^	0.97 ± 0.01 ^a^	0.97 ± 0.00 ^a^	0.97 ± 0.00 ^a^	0.97 ± 0.00 ^a^	0.97 ± 0.00 ^a^	0.97 ± 0.00 ^a^	0.97 ± 0.00 ^a^
0.12 g/L LO	0.97 ± 0.00 ^a^	0.97 ± 0.00 ^a^	0.97 ± 0.00 ^a^	0.97 ± 0.00 ^a^	0.97 ± 0.00 ^a^	0.97 ± 0.00 ^a^	0.97 ± 0.00 ^a^	0.97 ± 0.00 ^a^
0.6 g/L LO	0.97 ± 0.00 ^a^	0.97 ± 0.00 ^a^	0.97 ± 0.00 ^a^	0.97 ± 0.00 ^a^	0.97 ± 0.00 ^a^	0.97 ± 0.00 ^a^	0.97 ± 0.00 ^a^	0.97 ± 0.00 ^a^

^a^ Means with columns with the same letter (a) are not significantly different (*p* > 0.05) between treatments. ^1^ LO = lactose oxidase; *n* = 8.

**Table 4:** pH (±SD) of pasteurized queso fresco treated with lactose oxidase and sodium thiocyanate in the milk used for making the cheese during storage at 6 °C.

	Time (Days)							
Treatment ^1^	0	1	2	4	7	14	21	28
Control	6.32 ± 0.08 ^a^	6.34 ± 0.04 ^a^	6.36 ± 0.01 ^a^	6.42 ± 0.07 ^a^	6.41 ± 0.10 ^a^	6.25 ± 0.10 ^a^	6.34 ± 0.04 ^a^	6.33 ± 0.04 ^a^
0.12 g/L LO-TCN	6.30 ± 0.03 ^a^	6.29 ± 0.02 ^a^	6.24 ± 0.02 ^a^	6.13 ± 0.15 ^b^	5.95 ± 0.11 ^b^	5.88 ± 0.05 ^b^	5.91 ± 0.18 ^b^	5.79 ± 0.03 ^b^
0.6 g/L LO-TCN	6.29 ± 0.04 ^a^	6.20 ± 0.05 ^a^	6.11 ± 0.01	5.96 ± 0.09 ^b^	5.77 ± 0.09 ^c^	5.86 ± 0.21 ^b^	5.54 ± 0.07 ^c^	5.45 ± 0.14 ^c^

^a, b, c^ Means within a column with different letters are significantly different (*p* < 0.05) between treatments. ^1^ LO-TCN = lactose oxidase; TCN = sodium thiocyanate; *n* = 8.

**Table 5:** aW (±SD) of pasteurized queso fresco treated with lactose oxidase and sodium thiocyanate in the milk used for making the cheese during storage at 6 °C.

	Time (Days)							
Treatment ^1^	0	1	2	4	7	14	21	28
Control	0.97 ± 0.00 ^a^	0.98 ± 0.00 ^a^	0.97 ± 0.00 ^a^	0.97 ± 0.00 ^a^	0.97 ± 0.00 ^a^	0.97 ± 0.00 ^a^	0.97 ± 0.00 ^a^	0.97 ± 0.00 ^a^
0.12 g/L LO-TCN	0.97 ± 0.00 ^a^	0.97 ± 0.00 ^a^	0.98 ± 0.00 ^a^	0.97 ± 0.00 ^a^	0.97 ± 0.00 ^a^	0.97 ± 0.00 ^a^	0.97 ± 0.00 ^a^	0.97 ± 0.00 ^a^
0.6 g/L LO-TCN	0.97 ± 0.00 ^a^	0.97 ± 0.00 ^a^	0.98 ± 0.00 ^a^	0.97 ± 0.00 ^a^	0.97 ± 0.00 ^a^	0.97 ± 0.00 ^a^	0.97 ± 0.00 ^a^	0.97 ± 0.00 ^a^

^a^ Means with columns with the same letter (a) are not significantly different *(p* > 0.05) between treatments. ^1^ LO-TCN = lactose oxidase; TCN = sodium thiocyanate; *n* = 8.

**Table 6:** pH ± SD of queso fresco treated with thiocyanate and lactose oxidase on the surface of the cheese during storage at 6 °C.

	Time (Days)							
Treatment ^1^	0	1	2	4	7	14	21	28
Control	6.36 ± 0.01 ^a^	6.35 ± 0.03 ^a^	6.38 ± 0.01 ^a^	6.33 ± 0.04 ^a^	6.34 ± 0.02 ^a^	6.38 ± 0.12 ^a^	6.31 ± 0.03 ^a^	6.31 ± 0.03 ^a^
0.12 g/L LO	6.8 ± 0.02 ^a^	6.37 ± 0.02 ^a^	6.34 ± 0.01 ^a^	6.34 ± 0.01 ^a^	6.35 ± 0.03 ^a^	6.29 ± 0.05 ^ab^	6.24 ± 0.05 ^a^	6.23 ± 0.02 ^a^
0.6 g/L LO	6.38 ± 0.03 ^a^	6.45 ± 0.09 ^a^	6.35 ± 0.02 ^a^	6.35 ± 0.02 ^a^	6.30 ± 0.08 ^b^	6.19 ± 0.05 ^b^	6.10 ± 0.05 ^bc^	6.04 ± 0.01 ^b^
0.12 g/L LO-TCN	6.39 ± 0.02 ^a^	6.46 ± 0.07 ^a^	6.42 ± 0.01 ^a^	6.47 ± 0.06 ^b^	6.36 ± 0.06 ^a^	6.30 ± 0.06 ^ab^	6.25 ± 0.03 ^ab^	6.22 ± 0.02 ^a^
0.6 g/L LO-TCN	6.41 ± 0.02 ^a^	6.42 ± 0.05 ^a^	6.40 ± 0.10 ^a^	6.34 ± 0.01 ^a^	6.35 ± 0.10 ^a^	6.16 ± 0.05 ^b^	6.09 ± 0.05 ^c^	6.02 ± 0.03 ^b^

^a,b,c^ Means within a column with different letters are significantly different (*p* < 0.05) between treatments. ^1^ LO = lactose oxidase; TCN = thiocyanate; *n* = 8.

**Table 7:** aW ± SD of queso fresco treated with thiocyanate and lactose oxidase on the surface of the cheese during storage at 6 °C.

	Time (Days)							
Treatment ^1^	0	1	2	4	7	14	21	28
Control	0.97 ± 0.00 ^a^	0.97 ± 0.00 ^a^	0.97 ± 0.00 ^a^	0.97 ± 0.00 ^a^	0.98 ± 0.01 ^a^	0.97 ± 0.01 ^a^	0.97 ± 0.01 ^a^	0.98 ± 0.01 ^a^
0.12 g/L LO	0.98 ± 0.01 ^a^	0.97 ± 0.01 ^a^	0.97 ± 0.01 ^a^	0.96 ± 0.00 ^a^	0.97 ± 0.00 ^a^	0.97 ± 0.00 ^ab^	0.97 ± 0.00 ^a^	0.97± 0.00 ^a^
0.6 g/L LO	0.98 ± 0.01 ^a^	0.97 ± 0.00 ^a^	0.97 ± 0.00 ^a^	0.97 ± 0.00 ^a^	0.97 ± 0.00 ^a^	0.97 ± 0.00 ^a^	0.97 ± 0.00 ^a^	0.97 ± 0.00 ^a^
0.12 g/L LO-TCN	0.98 ± 0.00 ^a^	0.97 ± 0.01 ^a^	0.97 ± 0.00 ^a^	0.97 ± 0.00 ^a^	0.97 ± 0.01 ^a^	0.98 ± 0.01 ^a^	0.97 ± 0.00 ^a^	0.97 ± 0.00 ^a^
0.6 g/L LO-TCN	0.97 ± 0.00 ^a^	0.97 ± 0.01 ^a^	0.97 ± 0.00 ^a^	0.97 ± 0.00 ^a^	0.98 ± 0.00 ^a^	0.97 ± 0.00 ^a^	0.97 ± 0.01 ^a^	0.98 ± 0.01 ^a^

^a^ Means within a column with the same letters are not significantly different *(p >* 0.05) between treatments. ^1^ LO = lactose oxidase; TCN = thiocyanate; *n* = 8.

## Data Availability

The data represented in this study are available on request from the corresponding author.
